# Aerobic Training Intensity for Improved Endothelial Function in Heart Failure Patients: A Systematic Review and Meta-Analysis

**DOI:** 10.1155/2017/2450202

**Published:** 2017-02-27

**Authors:** M. J. Pearson, N. A. Smart

**Affiliations:** School of Science and Technology, University of New England, Armidale, NSW 2351, Australia

## Abstract

*Objective*. Flow-mediated dilation (FMD) is widely utilised to assess endothelial function and aerobic exercise improves FMD in heart failure patients. The aim of this meta-analysis is to quantify the effect of aerobic training intensity on FMD in patients with heart failure.* Background*. A large number of studies now exist that examine endothelial function in patients with heart failure. We sought to add to the current literature by quantifying the effect of the aerobic training intensity on endothelial function.* Methods*. We conducted database searches (PubMed, Embase, ProQuest, and Cochrane Trials Register to June 30, 2016) for exercise based rehabilitation trials in heart failure, using search terms exercise training, endothelial function, and flow-mediated dilation (FMD).* Results*. The 13 included studies provided a total of 458 participants, 264 in intervention groups, and 194 in nonexercising control groups. Both vigorous and moderate intensity aerobic training significantly improved FMD.* Conclusion*. Overall both vigorous and moderate aerobic exercise training improved FMD in patients with heart failure.

## 1. Introduction

Results of numerous studies and meta-analyses have now shown that exercise training is not only safe but is associated with a range of physiological, functional, and clinical benefits in patients with heart failure (HF) [1–3]. While exercise interventions in HF patients have utilised a range of training modalities, aerobic or endurance training is the most investigated and has been shown to improve a range of parameters in HF patients [1, 4], including endothelial function [5]. Endothelial dysfunction is associated with the pathogenesis and progression of HF [6] and flow-mediated dilation (FMD), a noninvasive assessment of endothelial function, has been shown to be predictive of deterioration and death [7] in HF patients. Aerobic exercise training improves endothelial dependent vasodilation primarily by improving nitic oxide (NO) bioavailability [8].

Despite a large number of exercise training studies it was not until 2011 that a consensus document by the Heart Failure Association (HFA) and European Association for Cardiovascular Prevention and Rehabilitation (EACPR) provided a detailed and comprehensive guideline for exercise training in HF patients [9]. However, while aerobic exercise is now a feature of cardiac rehabilitation guidelines around the world, training program characteristics still vary considerably and the focus of current and emerging research is on identifying the exercise modality, dose, and intensity that will deliver optimal benefits [10–13]. While all training characteristics will likely influence results to some degree, the role of exercise intensity in cardiac rehabilitation is considered a key issue [14]. As the pattern of blood flow and amount of shear stress [8] that occur during exercise may be related to the specific training characteristics, including training intensity, ascertaining an optimal training protocol is important.

A meta-analysis in HF patients by Ismail and colleagues (2013) [12] demonstrated that as exercise intensity increases the magnitude of change in VO_2 peak_ also increases. In addition, a considerable body of evidence is mounting in relation to aerobic intermittent or interval training in clinical populations including HF patients [15, 16], and more specifically in relation to high-intensity interval training (HIIT) [15] for improving a range of physiological, functional and clinical parameters, including vascular function [5].

While exercise intensity is associated with the magnitude of change in VO_2 peak_ in HF patients [12], the relationship between aerobic intensity and endothelial function is not clear. In healthy men, high-intensity exercise has been shown to increase oxidative stress reducing the bioavailability of NO and possibly negating the positive effect of exercise induced shear stress on endothelial function [17]. However, increases in antioxidant levels and greater improvements in FMD from HIIT compared to moderate intensity continuous training (MICT) in heart failure patients [5] suggest that intensity may have a role in the endothelial response to exercise in this population.

In a range of clinical populations both moderate [18] and high-intensity [19, 20] aerobic training have significantly improved FMD. A recent meta-analysis [21] across a diverse population reported a significant improvement in FMD from aerobic exercise and a significant dose-response relationship between intensity and FMD. In addition, Ramos and colleagues (2015) [22] examined the effects of high-intensity training, specifically HIIT compared to MICT across a diverse population, demonstrating HIIT to be more effective for improving FMD [22].

A number of aerobic exercise training studies have now investigated FMD in HF patients and therefore the primary aim of our paper was to conduct a systematic review and meta-analysis to investigate if training intensity reflects the magnitude of change in FMD.

## 2. Methods

### 2.1. Search Strategy

Potential studies were identified by conducting systematic searches of PubMed, Embase, CINAHL, SPORTDiscus, and the Cochrane Library of Controlled Trials up until 30 June 30, 2016. Searches included a mix of MeSH and free text terms related to the key concepts of heart failure, exercise training, endothelial function, and flow-mediated dilation. Additionally, systematic reviews, meta-analyses, and reference lists of papers were hand searched for additional studies. One reviewer (MJP) conducted the search; and full articles were assessed for eligibility by two reviewers (MJP and NAS). Two authors were contacted to provide additional information; one author did not respond and the second responded but was unable to provide any further details.

### 2.2. Study Selection

Randomised controlled trials and controlled trials of aerobic exercise training in heart failure patients with reduced ejection fractions (HFrEF) were included. Studies included in the review compare an aerobic training intervention to a no exercise or usual care control group or compared continuous aerobic training with interval or intermittent aerobic training. Only studies that measured endothelial function by flow-mediated dilation (FMD) measured via ultrasound reported as relative FMD% or absolute FMD (mm or *μ*m) in either the brachial or radial artery were included.

### 2.3. Data Extraction and Outcome Measures

Data were extracted by one reviewer (MJP). The primary outcome measure was flow-mediated dilation (FMD% or FMD absolute (mm)). Where FMD was reported as FMD% and FMD (mm), FMD% was utilised in the analysis.

### 2.4. Data Synthesis

Statistical analyses were performed using Revman 5.3 (The Nordic Cochrane Centre, Copenhagen, Denmark). The individual meta-analyses were completed for continuous data by using the change in the mean and standard deviation (SD). The primary outcome measure was FMD%. Where the change in mean and SD were not reported, the change in mean was calculated by subtracting the preintervention mean form the postintervention mean, and Revman 5.3 enabled calculations of SD using number of participants in each group, within or between group *p* values or 95% CI. In cases where exact *p* values were not provided, we used default values; for example, *p* < 0.05 becomes *p* = 0.049, *p* < 0.01 becomes *p* = 0.0099, and *p* = not significant becomes *p* = 0.051. Data not provided in main text or tables were extracted from figures. A random effects inverse variance was used with the effects measure of standardised mean difference (SMD). We utilised the widely accepted guideline for SMD interpretation [23], with 0.2 defined as small, 0.5 medium, and 0.8 as large. Where a study included multiple intervention groups and a control group, the sample size of the control group was divided by the number of intervention groups to eliminate over inflation of the sample size. We used a 5% level of significance and a 95% CI to report change in outcome measures. Aerobic intensity was defined and classified according to the ACSM (2011) [24]. Where prescribed intensity overlapped between two intensity classifications an additional analysis was conducted by reallocation of the studies to the alternative classification.

### 2.5. Heterogeneity and Publication Bias

Heterogeneity was quantified using the *I*^2^ test [25]. Values range from 0% (homogeneity) to 100% (highly heterogeneity) [25]. Egger tests and funnel plots [26] were provided to assess risk of publication bias.

### 2.6. Study Quality

Study quality was assessed by using the TESTEX, the tool for assessment of study quality and reporting, designed specifically for use in exercise training studies [27]. This is a 15-point scale that assesses study quality (maximum 5 points) and reporting (maximum 10 points). Two reviewers (MJP and NAS) conducted quality assessment.

## 3. Results

**Figure 1:**
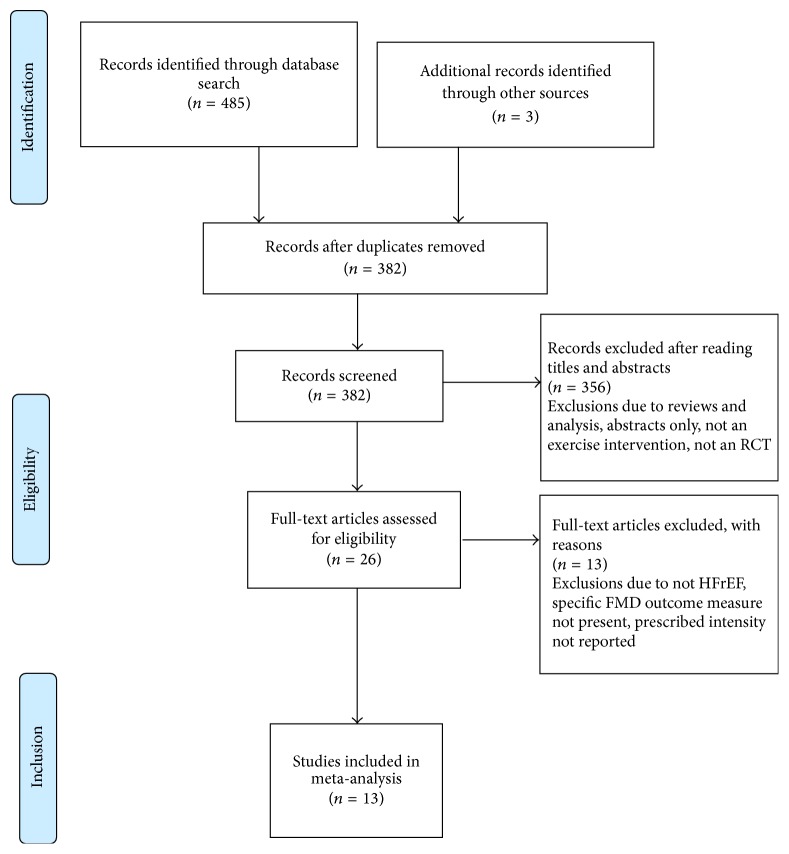
PRISMA flow diagram.

The initial search identified 485 manuscripts. After removal of duplicates and exclusion of articles based on abstract and title, 26 full-text articles remained for screening. Full screening resulted in 13 articles meeting the stated inclusion criteria (Figure 1 PRISMA statement). The aerobic exercise intervention characteristics of the 13 studies in the meta-analysis are included in Table 1. Details of full-text articles reviewed but excluded are provided in Supplementary Table  S1 in Supplementary Material available online at https://doi.org/10.1155/2017/2450202. Full participant details are provided in Supplementary Table  S2.

### 3.1. Study Characteristics

Thirteen [5, 28–39] studies provided a total of 458 participants diagnosed with HFrEF, 264 exercising participants, and 194 nonexercising control subjects. Twelve studies [5, 28–37, 39] included a usual care control group, of these, two studies [5, 28] included two different aerobic intervention groups. One study [38] did not include a control group and only compared intervention groups undertaking different aerobic exercise protocols. Ten studies [5, 29–33, 35–38] randomised participants, two studies were nonrandomised controlled trials [34, 39], and one study randomised participants between two exercise interventions but the control group was nonrandomised [28]. The average age of participants ranged between 49 ± 5 yrs and 76 ± 13 yrs and sex distribution was predominantly male. Brachial baseline FMD% ranged from ~3% to >7% and reported that baseline radial FMD% ranged from ~6% to >12% (Supplementary Table  S2).

### 3.2. Intervention Details

Intervention duration ranged from 4 weeks to 6 months, the frequency of sessions ranged from 2 days per week to daily, and the duration of exercise sessions ranged from 10 to 60 minutes. All studies performed an exercise test from which training intensity was prescribed and cycling was the most common mode of aerobic exercise. For pooled analysis, aerobic training intensity was classified according to ACSM (2011) [24]. The training protocol of four studies [5, 28, 34, 38] utilised interval/intermittent training and of these, three [5, 28, 34] utilised a training intensity deemed as high-intensity interval training (HIIT). Two [28, 38] studies employed short to moderate length intervals [40] and two [5, 34] utilised long length [40] intervals classified as a 4 × 4 HIIT protocol, but with different intensities. Seven [5, 28, 31, 34, 35, 37, 38] studies reported on how intensity was monitored, but only four [5, 28, 31, 34] studies reported actual or perceived (RPE) training intensity of participants and only one [32] reported actual energy expenditure (Supplementary Table  S3). Seven [5, 28, 30–32, 34, 37] studies reported session attendance percentages and 11 studies [5, 28–35, 37, 38] reported on the occurrence of any adverse events (Supplementary Table  S4). The assessment of FMD varied between studies (Supplementary  Table  S5) and 10 studies [5, 28–31, 33–35, 38, 39] assessed FMD in the Brachial Artery (BA), with the Radial Artery utilised in three studies [32, 36, 37].

## 4. Outcome Measures

### 4.1. Flow-Mediated Dilation (FMD)

#### 4.1.1. Moderate Aerobic Intensity versus Control

**Figure 2:**
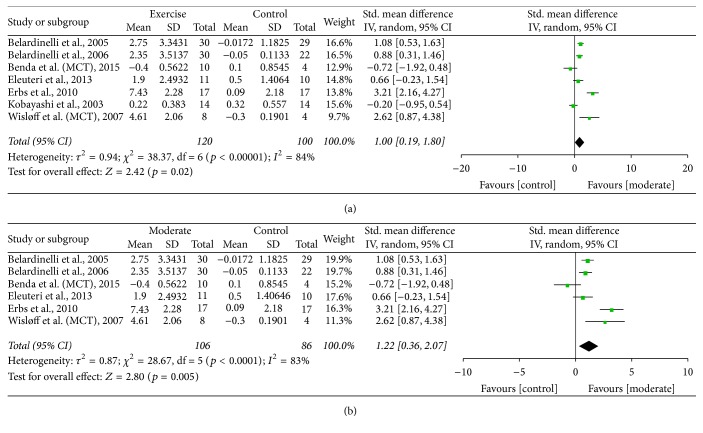
(a) FMD: moderate aerobic training versus control. (b) FMD: moderate aerobic training versus control (removal of Kobayashi study from moderate intensity).

Pooled data from seven studies [5, 28–32, 35] that utilised moderate intensity demonstrated a significant improvement in FMD, exercise versus control, SMD of 1.00 (95% CI 0.19 to 1.80, *p* = 0.02) (Figure 2(a)). The significance level increased with removal of the one non-RCT [28], SMD of 1.24 (95% CI 0.42 to 2.06, *p* = 0.003). One [35] study prescribed an intensity range that incorporates both the moderate and vigorous intensity definition, and removal of the study resulted in an increased SMD of 1.22 (95% CI 0.36 to 2.07, *p* = 0.005) (Figure 2(b)), which increased further with removal of the one non-RCT [28] [SMD of 1.53 (95% CI 0.72 to 2.35, *p* = 0.0002)].

#### 4.1.2. Vigorous Aerobic Intensity versus Control

**Figure 3:**
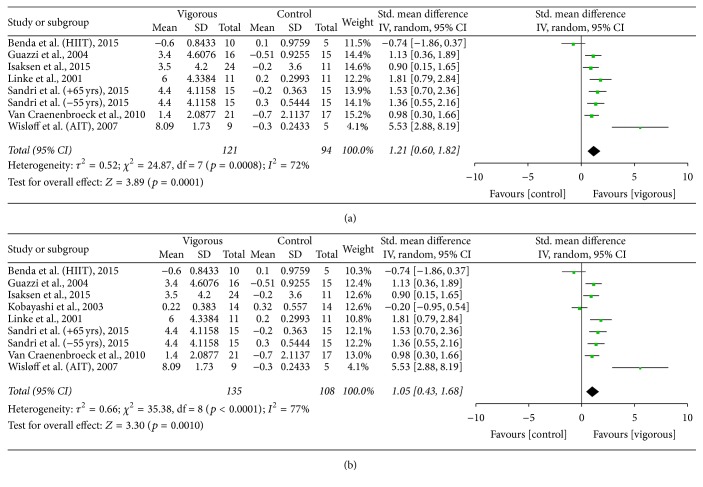
(a) FMD: vigorous aerobic training versus control. (b) FMD: vigorous aerobic training versus control (reallocation of Kobayashi from moderate to vigorous intensity).

Pooled data from seven studies [5, 28, 33, 34, 36, 37, 39] utilising vigorous intensity demonstrated a significant improvement in FMD, SMD of 1.21 (95% CI 0.60 to 1.82, *p* = 0.0001) (Figure 3(a)). Removal of the three non-RCTs [28, 34, 39] increased the significance, SMD of 1.69 (95% CI 0.97 to 2.40, *p* < 0.00001). Reclassification of the one [35] study that straddled both moderate and vigorous intensity decreased SMD to 1.05 (95% CI 0.43 to 1.68, *p* = 0.001) (Figure 3(b)); however with removal of the three non-RCTs [28, 34, 39] SMD increased to 1.43 (95% CI 0.56 to 2.30, *p* = 0.001).

#### 4.1.3. Aerobic Interval/Intermittent versus Continuous

Pooled data from three studies [5, 28, 38] demonstrated a nonsignificant change in FMD with interval training versus control; SMD of 0.56 (95% CI −0.49 to 1.61, *p* = 0.30) (Supplementary Figure  S1). With removal of the one non-RCT [28] the change in FMD increased but remained nonsignificant [SMD of 1.00 (95% CI −0.33 to 2.33, *p* = 0.14)]. One [38] study utilised a moderate intensity, with the remaining two studies [5, 28] utilising a high intensity. With removal of the one [38] moderate intensity study the result remained nonsignificant for HIIT versus continuous [SMD of 0.70 (95% CI −1.27 to 2.69, *p* = 0.49)].

#### 4.1.4. HIIT versus Control

Pooled data from three studies [5, 28, 34] that included a HIIT and control group, indicated a trend toward improvement with HIIT in FMD; however this was not significant, SMD of 1.80 (95% CI −0.69 to 4.29, *p* = 0.16) (Supplementary Figure  S2). Two [28, 34] of the three studies were however non-RCTs.

### 4.2. Endothelial-Independent Dilation

Six [28–30, 33, 34, 36] of the included studies noted the assessment of endothelial-independent vasodilation. Five studies [28–30, 33, 34] provided relative% change in arterial diameter, while one study [36] provided both absolute and relative% change. The endothelial-independent response did not differ significantly between exercise and control, SMD of −0.02 (95% CI −0.85 to 0.82, *p* = 0.97) (Supplementary Figure  S3).

### 4.3. Study Quality Assessment

The median TESTEX score was 9 (Supplementary Table  S6). While RCTs noted participant randomisation, specific details were lacking from the majority of studies. The majority of studies lost points in the areas of allocation concealment and activity monitoring in the control group.

### 4.4. Heterogeneity and Publication Bias

All analyses demonstrated moderate to high heterogeneity. Funnel plots demonstrated some evidence of publication bias.

## 5. Discussion

This work analysed the effects of aerobic training intensity on FMD in patients with chronic heart failure. Our primary finding shows that aerobic exercise training significantly improves endothelial function, assessed via FMD, in patients with heart failure. Our pooled data failed to find a significant change in endothelial-independent vasodilation, indicating that the improvement occurred at the level of the endothelium [41]. All but two [28, 35] of the studies included in our analysis found improvements in brachial or radial artery FMD. Interestingly, while Kobayashi et al. (2003) [35] failed to find any improvement in upper limb FMD they did report a significant improvement in lower limb artery FMD (posterior tibial artery).

Training intensity is considered a key component in determining optimal outcomes in cardiac rehabilitation [14] and our analysis demonstrated that both moderate and vigorous intensity, defined according to ACSM (2011) [24], significantly improved FMD of the brachial or radial artery. However, whether or not the magnitude of improvement increased with intensity remains unclear. As only four studies reported actual training intensities, our analysis of intensity was based on the prescribed training intensity for the exercise intervention. Whether or not vigorous or moderate intensity provided greater improvements in FMD was dependent upon the allocation of one [35] study, which prescribed a training intensity range that fell within both moderate and vigorous categories. Two analyses were therefore conducted to ascertain the effect of this study, and due to the nonsignificant finding of the study, reallocation demonstrated contrasting results. Based on the analysis we therefore cannot conclude that the magnitude of the improvement in FMD increases with intensity as was recently reported in the case of VO_2 peak_ by Ismail and colleagues [12]. Additionally, it is likely that the result would also vary depending on the actual definition or range of a particular intensity adopted, which varies between organization [24, 42], and whether or not the actual training intensities were as prescribed.

Since the impressive findings of Wisløff et al. (2007) [5] there has been an increased interest in aerobic intermittent/interval training and some guidelines [9] now advocate for this as a form of aerobic training in stable HF patients, although the actual prescribed intensity of the intervals still vary. We therefore conducted an analysis of HIIT compared to MICT. Our analysis of FMD indicated a trend toward interval or HIIT providing a greater improvement than MICT; however, the pooled results were not significant. Only the study of Wisløff et al. [5] demonstrated HIIT as significantly superior to MICT. However, only two [5, 38] of the three studies included in our analysis were RCTs and while the RCT of Smart and Steele (2012) [38] utilised interval training, the intensity of the intervals did not fall within the definition of HIIT [40]. Interval or intermittent training can be performed at any intensity; however, HIIT has been shown to invoke more significant improvements in VO_2 peak_ compared to MICT in HF patients [15, 16].

The broad definition of HIIT also means that a range of protocols are employed in both research and practice and a large number of variables can be manipulated in prescribing HIIT [43]. All three studies in our analysis of HIIT versus MICT utilised different protocols, with only Wisløff et al. (2007) [5] employing a long interval (4 × 4) protocol, which may account for some of the contrasting results between studies. Different interval/HIIT protocols may have different physiological responses and may impact the amount of shear stress [5, 22, 28]. For this reason a long HIIT protocol may be more effective [22]. Interestingly the participants in the Wisløff et al. [5] study also had lower baseline FMD% (<4%) than participants in the other two studies [28, 38] and therefore could provide a further explanation of the contrasting results, as lower baseline FMD% is one factor suggested as differentiating FMD responders from nonresponders [44]. Our nonsignificant finding is in contrast to the significant and superior improvement in FMD after HIIT compared to MICT in studies across a diverse population [22], although in CAD patients the recent SAINTEX-CAD study [45] reported significant improvements in FMD from HIIT and MICT with no difference between groups. Recently it was demonstrated in obese adults that HIIT and MICT may result in different vascular adaptations with HIIT improving FMD and MICT improving resting brachial diameter [46]. However, no studies in our review reported a significant change in resting arterial diameter after MICT. Interestingly, a recent meta-analysis that compared HIIT to MICT to investigate other clinical parameters in heart failure patients (not FMD) revealed mixed findings [13], while data from previous meta-analyses have shown HIIT more effective than MICT in improving VO_2 peak_  [12, 15].

In our pooled analysis of HIIT compared to no training, despite a trend toward HIIT, we failed to find a significant change in FMD. However, two of the three studies were non-RCTs [28, 34]. Of the three included studies, the non-RCT of Isaksen et al. (2015) [34] and RCT of Wisløff et al. (2007) [5] both reported a significant change in FMD in training groups after intervention with no change in controls, and interestingly both studies utilised a 4 × 4 HIIT protocol, which may be a more optimal protocol to improve vascular function [22]. Interestingly, a short duration HIIT interval (30 seconds work; 60 seconds rest) utilised by Anagnostakou et al. [47] in a comparison of HIIT to combined HIIT and resistance training failed to elicit a significant improvement in FMD in a HIIT only training group. However, FMD improved in a combined HIIT and resistance training group. Of particular interest is that, in the Isaksen et al. [34] study, while HR data was not stored for intensity analysis on any variables, they do note that, in a separate analysis on VO_2 peak_, the improvement in VO_2 peak_ was almost doubled in patients who reported an average RPE ≥ 16, and while no details are provided on FMD, one can question whether this may have occurred with FMD, indicating the role of intensity.

As there are still unanswered questions in relation to the role of endothelial dysfunction in the development and symptoms of HF patients with preserved ejection fractions [48] our analysis only included patients with reduced ejection fractions. Therefore our analysis cannot be generalised to HFpEF patients. Additionally, only minimal studies to date exist that have utilised aerobic training and investigated FMD. Kitzman and colleagues (2013) [49] failed to find any significant change in FMD following 16 weeks of high-intensity aerobic training (70%  VO_2 max_), while more recently Angadi et al. (2015) [50] in a relatively small, short duration (4 weeks) study compared HIIT and MICT and failed to find a significant change in FMD in either group. 

*Strengths and Limitations in the Systematic Review and Meta-Analysis*. To the best of our knowledge this is the first meta-analysis that provides analysis on aerobic training intensity and endothelial function in heart failure patients. The major limitation of the review is the high level of heterogeneity among studies. Differences in the methodological assessment of FMD and medication use may have contributed to the level of heterogeneity. Another limitation of the review is the classification of exercise intensity. We classified aerobic intensity according to the ACSM (2011) guidelines [24], which provides intensity ranges based on % HRR or VO_2_ reserve (VO_2_R), VO_2 max_, HR_max_, RPE, or Metabolic Equivalent of Task (METS). Over the years these ranges have changed which would change the classification of studies. Additionally, intensity ranges defined by other organizations [42] differ from the ACSM [24]. As the majority of studies did not report on the actual training intensities of the sessions, whether or not the mean training intensity was firstly within the prescribed intensity range for the duration of the intervention and secondly whether the mean training intensity was closer to the upper or lower end of the prescribed ranges could not be ascertained. We were unable to conduct an analysis according to different intensity domains and thresholds, as opposed to ranges, as suggested by Mezzani et al. (2012) [14], as the relevant information could not be extracted from all studies. In regard to data pooling, we measured the difference between preintervention and postintervention means; however, in cases where exact *p* values, within groups or between groups, or 95% CI were not available, default values for *p* were utilised and this may introduce errors. Additionally, data from some studies was extracted from figures; this in itself has the potential to introduce errors.

## 6. Conclusion

This meta-analysis found that both vigorous and moderate aerobic exercise training improves endothelial function, assessed by FMD, in heart failure patients with reduced ejection fractions. Future studies investigating FMD responses to different training intensities including high-intensity training protocols will further assist in providing more evidence as to optimal aerobic training intensity prescription to elicit superior improvements in endothelial function as well as other physiological and clinically relevant endpoints.

## Supplementary Material

Online supplementary material contains supplementary figures S1, S2 and S3 as referrred to in section 4.1.3, 4.1.4 and 4.2 of the review. Supplementary material also contains details of excluded studies, additional participant and intervention characteristics and a table of assessment of study quality.

## Figures and Tables

**Table 1:** Aerobic exercise characteristics of studies included in meta-analysis.

Study	Study design	Sample size (completed/analysed)	Intervention duration (weeks)	Training modality	Frequency (per wk.)	Session duration	Prescribed exercise intensity
Benda et al. (2015) [28]	Non-RCT^(1)^	29	12	Cycle	2	35 min (HIIT) 30 min (CT) *plus* 10 min warm-up, 5 min cooldown each group	*HIIT*: 10 × 1 min @ 90% max. workload (RPE 15–17) separated by 2.5 min @ 30% max. workload *CT*: @60–75% max. workload (RPE 12–14) warm-up @ 40% max. Workload & cooldown @ 30% max. workload

Belardinelli et al. (2006) [29]	RCT	52	8	Cycle	3	40 min *plus *15 min warm-up stretch, 5 min cooldown	60% VO_2 peak_

Belardinelli et al. (2005) [30]	RCT	59	8	Cycle	3	40 min *plus* 15 min warm-up stretch, 5 min cooldown	60% VO_2 peak_

Eleuteri et al. (2013) [31]	RCT	21	12	Cycle	5	30 min *plus* 5 min warm-up, 5 min cooldown	HR & power @ VAT (cycle @ 60 RPM) (VAT ~ 60% VO_2 max_)^1^

Erbs et al. (2010) [32]	RCT	34	12	Cycle 1 × GS^*∗*^	Daily +1 GS wk.	20–30 min (*plus* 1 × 60 min GS/wk.)	HR @ 60% VO_2 max_

Guazzi et al. (2004) [33]	RCT	31	8	Cycle	4	30 min *plus* 5 min warm-up, 5 min cooldown	60% HRR wk. 1-2, ↑ 80% HRR @ wk. 3

Isaksen et al. (2015) [34]	Non-RCT	35	12	Cycle or treadmill	3	25 min *plus* 15 min warm-up, 5 min cooldown, 15 min strength/stretch	4 × 4 HIIT @ 85% HR_max_ (~RPE 15–17) separated by 3 min recovery @ 60–70% HR_max_, warm-up @ 60–70% HR_max_

Kobayashi et al. (2003) [35]	RCT	28	12	Cycle	2-3 (2x day)	2 × 15 min session/day (30 min/day total)	HR @ VAT (~60–70% VO_2 max_)

Linke et al. (2001) [36]	RCT	22	4	Cycle	daily (6x per day)	10 min (60 min/day total)	70% VO_2 peak_

Sandri et al. (2015) [37]	RCT	60	4	Cycle 1 × GS^*∗*^	5 (4x per weekday)	15–20 min (~60 min/day total) *plus* 5 min warm-up and cooldown (*plus* 1 × 60 min GS per/wk.)	70% of symptom limited VO_2 max_

Smart and Steele (2012) [38]	RCT	23	16	Cycle	3	60 min (INT) 30 min (CONT)	*INT*: work : rest (60 s : 60 s) @ 60–70% VO_2 peak_*CT*: 60–70% VO_2 peak_ (cycle @ 60 RPM)

Van Craenenbroeck et al. (2010) [39]	Non-RCT	38	26	Ambulatory base	3	60 min	90% HR @ respiratory compensation point

Wisløff et al. (2007) [5]	RCT	26	12	Treadmill/ home walking	3	28 min (AIT) *plus* 10 min warm-up 47 min (MICT)	*AIT*: 4 min × 4 @ 90–95% HR_max_, separated by 3 min @ 50–70% HR_max_, *MICT*: @ 70–75% HR_max_

AIT: aerobic interval training, Con: control, CT: continuous training, GS: group session, HIIT: high intensity interval training, HR: heart rate, HR_max_: maximum heart rate, HR_peak_: peak heart rate, HRR: heart rate reserve, MIACT: moderate intensity aerobic training, MICT: moderate continuous training, non-RCT: nonrandomised controlled trial, RCT: randomised controlled trial, RPE: rating of perceived exertion, RPM: revolutions per minute, VAT: ventilatory anaerobic threshold, VO_2 peak_: peak oxygen uptake, and VO_2 max_: maximal oxygen uptake. ^(1)^Two exercise groups randomised, but control group not randomised. ^1^VO_2_ @ VT/VO_2 peak_ = 8.8/14.8 = 59.5% of VO_2 peak_. ^*∗*^1 group session per week composed of walking, calisthenics, and ball games.
