# Supplementary material for: Aerobic Training Intensity for Improved Endothelial Function in Heart Failure Patients: A Systematic Review and Meta-Analysis
Source: Cardiol Res Pract. 2017 Feb 27;2017:2450202. doi: 10.1155/2017/2450202 (PMC5350392; doi:10.1155/2017/2450202)
Supplement: Supplementary file 1 — Online supplementary material contains supplementary figures S1, S2 and S3 as referrred to in section 4.1.3, 4.1.4 and 4.2 of the review. Supplementary material also contains details of excluded studies, additional participant and intervention characteristics and a table of assessment of study quality. [file 2450202.f1.docx]

Supplementary Data File – Figures and Tables

Supplementary Fig. S1 FMD Interval vs. continuous


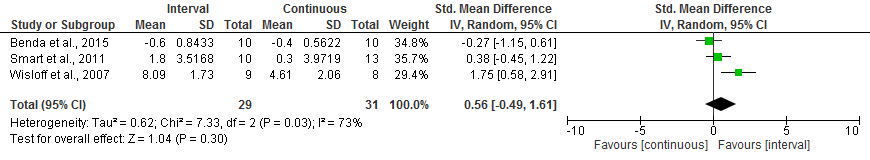


Supplementary Fig. S2 FMD HIIT vs. control


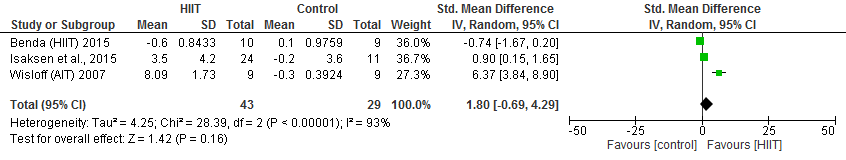


Supplementary Fig. S3 NMD Aerobic vs. Control

**
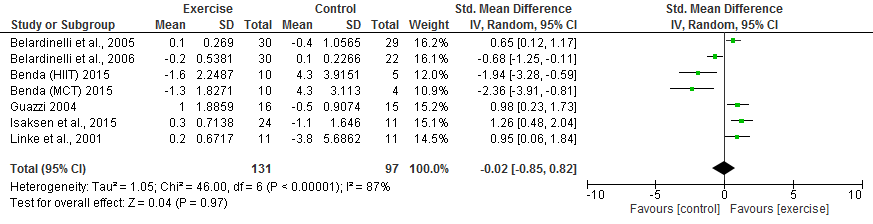
**

**Supplementary Table S1**. Studies reviewed but excluded with reason

| Study | Reason for Exclusion |
| --- | --- |
| Aksoy (2015) | No ultrasound FMD measurement, endothelial damage assessed via endothelial biomarkers (e.g., VCAM) |
| Anagnostakou (2011) | Comparison of Combined trained to interval training, no non-exercise control group |
| Angadi (2015) | Heart Failure preserved ejection fraction patients |
| Belardinelli (2008) | Possible data crossover from already included studies. Unable to confirm with Author. |
| Deftereos (2010) | Comparison of FES to conventional cycling, no control group. |
| Giannattasio (2001) | No details of prescribed exercise intensity |
| Hambrecht (1998) | Assessment of Femoral Artery and via Intra-arterial infusion to assess endothelial dilation |
| Kitzman (2013) | Heart Failure preserved ejection fraction patients |
| Ozasa (2011) | Endothelial function assessed via RH-PAT (Plethysmography technique) |
| Parnell (2002) | No measure of Ultrasound FMD to RH, only FBF to ACh |
| Laurent (2009) | No FMD measurement, assessment of NO metabolites only in Land vs. Water exercise. |
| Maiorana (2011) | No measure of FMD noted in study, only BA diameter |
| Mezzani (2013) | Same Study as Eleuteri (already include) |

**Supplementary Table S2.** Additional participant characteristics

| **Author** | **Group** | ***n=*** | **Age (yrs.)** | **% Male** | **LVEF%** | **Baseline**  **VO_2max_** | ***Baseline FMD%*** | **Aetiology** | ***NYHA Class*** |
| --- | --- | --- | --- | --- | --- | --- | --- | --- | --- |
| Benda (2015) | Ex 1: HIIT  Ex 2: MICT  C | 10  10  9 | 63±8  64±8  67±7 | 90%  100%  56% | 37±6  38±6  40±11 | 19.1±4.1  21.0±3.4  17.4±5.8 | 5.3±2.6%  5.2±2.5%  5.3±2% | Ischemic & non-ischemic | II & III |
| Belardinelli (2006) | Ex  C | 30  22 | 55±14  53±15 | 100%  100% | 30±7  34±8 | 14.8±2.5  14.7±2.5 | ~4%  ~4% | Previous MI, stenting & CABG | II & III |
| Belardinelli (2005) | Ex  C | 30  29 | 56±15  58±15 | 100%  100% | 39±6  28±5 | 16.8±3.7  15.9±1.5 | 2.29±1.13%  ~3% | Ischemic & idiopathic | II & III |
| Eleuteri (2013) | Ex  C | 11  10 | 66±2  63±2 | 100%  100% | 28±2  30±2 | 14.8±0.7  16.7±0.4 | 5.1±0.7%  7.7±1.4% | Ischemic & idiopathic CM | II |
| Erbs (2010) | Ex  C | 17  17 | 60±11  62±10 | 100%  100% | 24±5  25±4 | 15.3±3.3  15.4±3.8 | 6.1±2.5% (RA)  5.9±2.5% (RA) | Ischemic & DCM | III(b) |
| Guazzi (2004) | Ex  C | 16  15 | 52±5  54±4 | 100%  100% | 34±3  36±4 | ~17.0  ~16.3 | 4.8±0.4%  ~4.5% | Ischemic & DCM | II & III |
| Isaksen (2015) | Ex  C | 24  11 | 65±9  69±9 | 88%  100% | 38±11  30±8 | 17.4±4.6  16.9±2.8 | 6.41±3.44%  7.15±4.5% | Ischemic & DCM | I, II & III |
| Kobayashi (2013) | Ex  C | 14  14 | 55±2  62±2 | 86%  57% | 29±2  33±2 | 18.0±1.3  13.7±0.9 | 4.34±0.45%  4.19±0.45% | Ischemic & DCM | II & III |
| Linke (2001) | Ex  C | 11  11 | 58±2  59±3 | 100%  100% | 26±3  24±2 | NR | 11.3±2% (RA)  11.7±1% (RA) | Ischemic & DCM | II & III |
| Sandri (2015) | Ex 1 (<55yrs)  C 1 (<55yrs)  Ex 2 (>65yrs)  C 2 (>65yrs) | 15  15  15  15 | 50±5  49±5  72±4  72±3 | 80%  87%  80%  80% | 27±6  28±5  29±6  28±6 | 13.3±1.6  13.6±1.3  12.9±1.4  13.1±1.5 | 11.3±2.5% (RA)  11.7±2.0% (RA)  10.5±1.5% (RA)  11.2±1.4% (RA) | Ischemic & DCM | II & III |
| Smart (2012) | Ex 1: INT  Ex 2: MICT | 10  13 | 59±11  63±9 | 80%  100% | 27±8  30±8 | 12.6±6.5  12.4±2.5 | 7.4±5.5%  8.0±5.2% |  | II & III |
| Van Craenenbroeck (2010) | Ex  C | 21  17 | 61±2  63±3 | 86%  71% | 27±2  31±2 | 18.3±1.4  21.3±2.1 | 5.1±0.3%  5.9±0.6% | Ischemic & DCM | II |
| Wisloff (2007) | Ex: AIT  Ex: MICT  C | 9  8  9 | 77±9  74±12  76±13 | 78%  78%  67% | 28±7  33±5  26±8 | 13.0±1.6  13.0±1.1  13.2±1.9 | ~3.5%  ~3.7%  ~3.8% | Ischemic post infarct on β-blockers |  |

AIT: aerobic interval training, DCM: dilated cardiomyopathy, CABG: coronary artery bypass graft, Ex: exercise training, C: control, CT: continuous training, FMD: flow-mediated dilation, HIIT: high intensity interval training, INT: intermittent training, LVEF: left ventricular ejection fraction, MI: myocardial infarction, MICT: moderate intensity continuous training, NR: not reported, NYHA: New York Heart Association, RA: radial artery

**Supplementary Table S3.** Intensity Characteristics of Included Studies

| **Study** | **Intensity Prescribed** | **Monitoring of Intensity** | **Actual Training Intensity Reported** | **Energy Expenditure recorded or calculated** |
| --- | --- | --- | --- | --- |
| Belardinelli (2005) | 60% VO_2peak_ | Not Reported | Not Reported | Not Reported |
| Belardinelli (2005) | 60% VO_2peak_ | Not Reported | Not Reported | Not Reported |
| Benda (2015) | MICT – 60-75% max workload (RPE 12-14)  HIIT – 90% max workload  (RPE 15-17) | Intensity monitored using RPE  - 12-14 for MICT  - 15-17 for HIIT | MICT:  - Actual WL 66±5% of max WL  - Actual HR 81±7% HR_max_  - Reported RPE 13±1  HIIT:  - Actual WL 102±7% of max WL  - Actual HR 83±9% HR_max_  - Reported RPE 14±1 | Not Reported |
| Eleuteri (2013) | HR @ VT  (mean VT~60% VO_2peak_)^1^ | Intensity monitored using portable electrocardiograph  CPET repeated after  6 weeks to adjust training  intensity. | Actual Heart rate achieved = 102±4% of prescribed | Not Reported |
| Erbs (2010) | HR @ 60% VO_2max_ | Not Reported | Not Reported | 650kCal/week |
| Guazzi (2004) | 60-80% HRR | Not Reported | Not Reported | Not Reported |
| Isaksen (2015) | 85% HR_max_  (RPE 15-17) | Intensity monitored using HR monitors | HR data not stored for intensity analysis  Mean RPE 15.3±1.4  (50% patients RPE ≥16) | Not Reported |
| Kobayashi (2003) | HR @ VT (60-70% VO_2max_)  (mean VT~67%VO_2max_)^2^ | Intensity monitored using telemetry to monitor HR.  Exercise speed was adjusted in each to maintain the HR equivalent to the VT.  RPE used when difficult to assess HR- exercise speed regulated within the rating of 13 RPE | Not Reported | Not Reported |
| Linke (2001) | 70% VO_2peak_ | Not Reported | Not Reported | Not Reported |
| Sandri (2015) | 70% VO_2max (Symptom limited)_ | Workloads were adjusted to a HR so that 70% of the symptom-limited VO_2max_ was reached  No adjustment to intensity as was a 4 week training period. | Not Reported | Not Reported |
| Smart (2012) | 60-70% VO_2peak_ | Exercise intensity was uptitrated by 2 to5 W⁄ week.  In patients in paced rhythm or experiencing frequent ectopy RPE was used with a target RPE of 3 to 5 (moderate to hard) on the modified Borg scale. | Not Reported | Not Reported |
| Van Craenenbroeck (2010) | 90% HR @ RCP | Not Reported | Not Reported | Not Reported |
| Wisloff (2007) | AIT - 90-95% HR_max_  MICT - 70-85% HR_max_ | Intensity monitored using HR monitor and RPE during and after sessions.  Speed and incline of the treadmill adjusted to ensure training carried out at the assigned HR. | Intensity recorded as km/hr on treadmill, inclination and RPE:  - AIT= RPE 17±1 & MICT =RPE 12±1 | Not Reported |

1.^.^ VO_2_ @ VT/VO_2peak_ = 8.8/14.8 =59.5% of VO_2peak,_  2. VO_2_ @ VT/VO_2peak_ = 12.0/18.0 = 66.7% of VO_2peak_, AIT: Aerobic interval training CPET: cardiopulmonary exercise test, HIIT: high intensity interval training, HR: heart rate, HRR: heart rate reserve, HR_max_: maximum heart rate, INT: intermittent training, MICT: moderate-intensity continuous training, RCP: respiratory compensation threshold, RPE: ratings of perceived exertion, VO_2peak_: peak oxygen uptake, VT: ventilatory threshold, WL: workload

**Supplementary Table S4.** Intervention Adherence and Adverse Events

| **Study** | **Intervention Attendance** | **Adverse Events** |
| --- | --- | --- |
| Benda (2015) | 100% (missed sessions rescheduled) | 1 dropout each training group due to Progression HF  1 dropout each group due to musculoskeletal complaints  Nil other training related events |
| Belardinelli (2005) | 88% | Nil Adverse Events |
| Belardinelli (2006) |  | Nil Adverse Events |
| Eleuteri (2013) | Non-adherence <1% | Nil Adverse Events |
| Erbs (2010) | ~90% compliance | 1 Sudden Cardiac Death (Control) |
| Guazzi (2004) |  | Nil Adverse Events |
| Isaksen (2015) | 98% (average) (n=20 100%)  No patient completed <75% | No symptomatic arrhythmias during AIT  1 patient in control and 1 in training group experienced one episode of anti-tachycardia pacing (ATP), but not during or after the session  I patient complained of dizziness during two AIT sessions due to hypotension  1 patient in AIT group has a non-sustained supraventricular tachycardia during initial ergospirometry test  No other adverse events during intervention period |
| Kobayashi (2003) |  | Nil Adverse Events |
| Linke (2001) |  |  |
| Sandri (2015) | 100%^*^ | Nil Adverse Events^*^ |
| Smart (2012) | NR – Good adherence noted | Nil Adverse Event |
| Van Craenenbroeck (2010) |  |  |
| Wisloff (2007) | AIT = 92±2%  MCT =95±3% | Nil Adverse Events related to training  1 cardiac death in MCT group - unrelated to training |

* Reported in Sandri M, Kozarez I, Adams V, et al. Age-related effects of exercise training on diastolic function in heart failure with reduced ejection fraction: The Leipzig Exercise Intervention in Chronic Heart Failure and Aging (LEICA) diastolic dysfunction study. Eur Heart J 2012, 33: 1758–176

**Supplementary Table S5.** Summary of Flow-mediated dilation (FMD) assessment via Reactive Hyperaemia (RH)

| **Author** | **Artery** | **Cuff Position**  **(upper limb)** | **Cuff Pressure (mmHg)** | **Occlusion duration (minutes)** | **Notes on Guidelines, measurements** |
| --- | --- | --- | --- | --- | --- |
| Belardinelli (2006) | BA | Wrist | 240 | 4.5 | According to Guidelines (Corretti 2002) |
| Belardinelli (2005) | BA | Wrist | 240 | 4.5 | According to Guidelines (Corretti 2002) |
| Benda (2015) | BA |  |  |  | According to Guidelines (Thijssen 2011) |
| Erbs (2010) | RA |  | 50 above systolic | 5 |  |
| Eleuteri (2013) | BA | Forearm |  | 5 | According to Guidelines (Corretti 2002) |
| Guazzi (2004) | BA | Forearm | 50 above systolic | 5 | Guidelines - BARTF (Corretti 2002) |
| Isaksen (2015) | BA |  |  |  | Guidelines BARTF (Corretti 2002) |
| Kobayashi (2003) | BA | Forearm | 200 | 5 |  |
| Linke (2001) | RA |  | 50 above systolic | 5 |  |
| Sandri (2015) | RA |  | 50 above systolic | 5 | As described in Linke (2001) |
| Smart (2012) | BA | Forearm | 250 | 4.5 |  |
| Van Craenenbroeck (2010) | BA | Forearm | 200 (or 50 above systolic) | 4 | According to Guidelines (Corretti 2002) |
| Wisloff (2007) | BA | Upper Arm | 250 | 5 | According to Guidelines (Corretti 2002) |

**Supplementary Table S6**. Assessment of study quality and reporting using TESTEX

| Study | Eligibility Criteria specified | Randomisation  Details Specified | Allocation concealed | Groups similar at baseline | Assessors blinded | Outcomes measures assessed >85% participants# | Intention to treat analysis | Reporting between group statistical comparison* | Point measures & measures of variability | Activity monitoring in control group | Relative exercise intensity constant | Exercise volume & Energy expenditure | Overall TESTEX (/15) |
| --- | --- | --- | --- | --- | --- | --- | --- | --- | --- | --- | --- | --- | --- |
| ***RCTs*** |  |  |  |  |  |  |  |  |  |  |  |  |  |
| Belardinelli (2006) | 1 | 0 | 0 | 1 | 0 | 2 | 1 | 2 | 1 | 0 | 0 | 0 | 8 |
| Belardinelli (2005) | 1 | 0 | 0 | 1 | 0 | 3 | 1 | 2 | 1 | 0 | 0 | 0 | 9 |
| Eleuteri (2013) | 1 | 0 | 0 | 1 | 1 | 3 | 1 | 0 | 1 | 0 | 1 | 1 | 10 |
| Erbs (2010) | 1 | 1 | 1 | 1 | 1 | 3 | 0 | 2 | 1 | 0 | 0 | 0 | 11 |
| Guazzi (2004) | 1 | 1 | 0 | 1 | 1 | 1 | 0 | 2 | 1 | 1 | 0 | 0 | 9 |
| Kobayashi (2003) | 1 | 0 | 0 | 1 | 1 | 2 | 1 | 1 | 1 | 0 | 0 | 0 | 8 |
| Linke (2001) | 1 | 0 | 0 | 1 | 0 | 1 | 1 | 2 | 1 | 0 | 0 | 0 | 7 |
| Sandri (2015) | 1 | 1 | 1 | 1 | 1 | 3 | 1 | 2 | 1 | 0 | 0 | 0 | 12 |
| Smart (2012) | 1 | 0 | 0 | 1 | 0 | 2 | 1 | 2 | 1 | NA | 1 | 0 | 9 |
| Wisloff (2007 | 1 | 1 | 0 | 1 | 1 | 3 | 0 | 2 | 1 | 0 | 1 | 0 | 12 |
|  |  |  |  |  |  |  |  |  |  |  |  |  |  |
| ***Non- Randomised*** |  |  |  |  |  |  |  |  |  |  |  |  |  |
| Benda (2015) | 1 | 0 | 0 | 1 | 0 | 2 | 0 | 1 | 1 | 0 | 1 | 1 | 8 |
| Isaksen (2015) | 1 | 0 | 0 | 1 | 1 | 3 | 0 | 2 | 1 | 0 | 0 | 0 | 9 |
| Van Craenenbroeck (2010) | 1 | 0 | 0 | 1 | 1 | 1 | 1 | 2 | 1 | 0 | 0 | 0 | 8 |

Key: total out of 15 points. Legend: #three points possible—one point if adherence >85%, one point if adverse events reported, one point if exercise attendance is reported. *Two points possible—one point if primary outcome is reported, one point if all other outcomes reported. TESTEX, Tool for the assessment of Study quality and reporting in Exercise. 0 awarded if no mention was made of this criteria or if it was unclear
